# Supplementary material for: Living to the High Extreme: Unraveling the Composition, Structure, and Functional Insights of Bacterial Communities Thriving in the Arsenic-Rich Salar de Huasco Altiplanic Ecosystem
Source: Microbiol Spectr. 2021 Jun 30;9(1):10.1128/spectrum.00444-21. doi: 10.1128/spectrum.00444-21 (PMC8552739; doi:10.1128/spectrum.00444-21)
Supplement: SUPPLEMENTAL FILE 1 — Fig. S1 to S3; Tables S1, S2, S4, S6, and S8; and captions to Tables S3, S5, and S7. Download SPECTRUM00444-21_Supp_1_seq11.pdf, PDF file, 0.6 MB [file spectrum00444-21_supp_1_seq11.pdf]

SUPPLEMENTARY MATERIAL

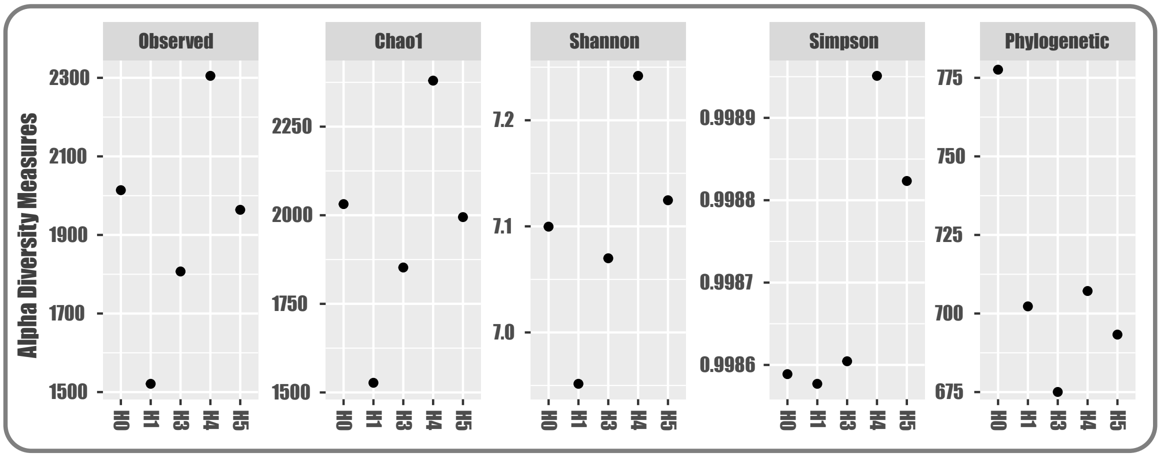

Supplementary Figure S1. Alpha diversity indexes for the five SH communities.

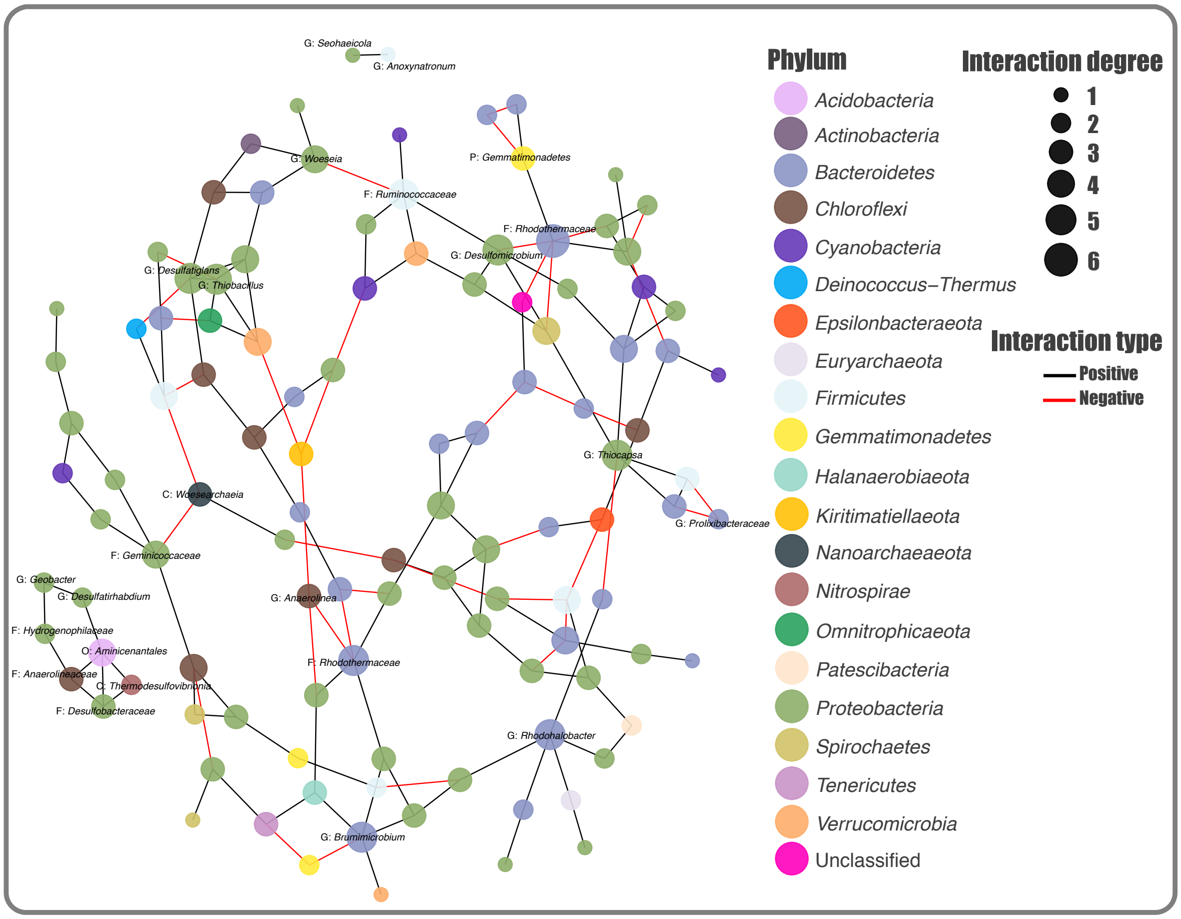

Supplementary Figure S2. Co-occurrence network analysis of the SH bacterial communities. The size of each node (representing ASVs) is proportional to the number of connections (degrees), the color of the edges connecting nodes represents the interaction type, the node color indicates the taxonomic affiliation at the phylum level and node labels are at the lowest available taxonomic classification.

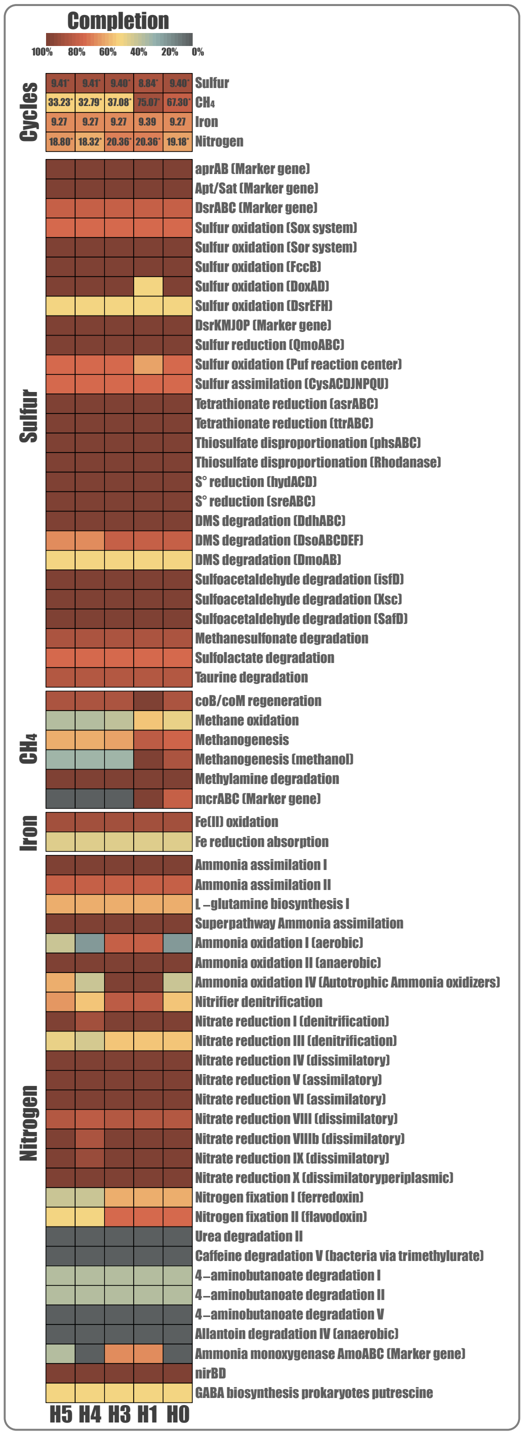

Supplementary Figure S3. MEBS analysis heatmap displaying the completeness of N, Fe, S and CH_4 pathways, as a whole (first top section) and for each particular pathway (bottom four sections). The color gradient shows the percentage of completion for each pathway (from lowest to highest) and the values shown in the top section represent the corresponding MEBS score (* FDR ≤ 0.01).

Supplementary Table S1. Physicochemical parameters and measured characteristics of the five SH sampled sites, as published previously in Castro-Severyn et al., 2020 [8].

Site	pH	Altitude (masl)	Sampling Temperature (ºC)	As [mg/kg]	Salinity (%)	Conductivity (mS)	Suspended Solids (gr/L)	Coordinates
H0	8.8	3792	14.6	9	11.9	6.082	3.006	20º15'48.8"S 68º52'28.4"W
H1	9.4	3785	14	16.3	8.1	4.076	2.039	20º16'27.7"S 68º53'3"W
H3	8.5	3783	19.9	49.2	2.2	1.122	0.560	20º16'59.2"S 68º53'16.7"W
H4	8.4	3787	18.8	155	77.2	38.20	19.40	20º17'40.9"S 68º53'17.3"W
H5	9.2	3781	24.3	321	84.5	42.12	21.12	20º18'37"S 68º52'42"W

Supplementary Table S2. Relative abundance of all detected phyla in the five SH communities.

Phylum	Proportion (%)				
	H0	H1	H3	H4	H5
Proteobacteria	29.067	40.343	36.431	41.857	33.732
Bacteroidetes	19.051	11.293	26.303	25.570	33.257
Cyanobacteria	33.987	11.488	6.932	16.001	14.524
Deinococcus-Thermus	2.038	0.078	5.236	5.286	2.901
Chloroflexi	1.176	8.489	6.072	1.614	2.996
Actinobacteria	0.647	4.050	3.712	2.539	2.313
Gemmatimonadetes	1.137	1.752	5.949	0.169	2.920
Patescibacteria	2.940	2.687	2.409	0.989	1.858
Verrucomicrobia	1.313	1.986	1.131	1.393	1.100
Firmicutes	2.646	1.051	1.377	0.586	0.626
Euryarchaeota	0.647	1.051	1.229	1.302	0.891
Spirochaetes	1.999	1.713	0.172	0.169	0.436
Planctomycetes	0.764	2.414	1.278	0.000	0.265
Kiritimatiellaeota	0.921	0.701	0.344	0.404	0.417
Acidobacteria	0.039	2.687	0.688	0.000	0.133
Tenericutes	0.353	0.156	0.049	0.833	0.190
Epsilonbacteraeota	0.274	0.701	0.000	0.286	0.683
Nanoarchaeaeota	0.039	2.843	0.049	0.052	0.076
Halanaerobiaeota	0.000	0.000	0.074	0.703	0.284
Fibrobacteres	0.294	0.389	0.049	0.000	0.000
Omnitrophicaeota	0.039	0.818	0.000	0.000	0.000
Latescibacteria	0.000	0.234	0.123	0.000	0.152
LCP-89	0.039	0.156	0.049	0.052	0.000
Lentisphaerae	0.000	0.078	0.147	0.052	0.000
Caldiserica	0.176	0.078	0.000	0.000	0.000
Chlamydiae	0.000	0.000	0.000	0.078	0.095
Cloacimonetes	0.176	0.000	0.000	0.000	0.038
Crenarchaeota	0.000	0.428	0.000	0.000	0.000
Nitrospirae	0.000	0.428	0.000	0.000	0.000
WS1	0.059	0.000	0.074	0.065	0.000
Hydrogenedentes	0.098	0.078	0.074	0.000	0.000
TA06	0.000	0.350	0.000	0.000	0.000
Rokubacteria	0.000	0.234	0.000	0.000	0.000
Altiarchaeota	0.000	0.195	0.000	0.000	0.000
BRC1	0.039	0.000	0.000	0.000	0.038
Fusobacteria	0.000	0.156	0.000	0.000	0.000
WS4	0.000	0.156	0.000	0.000	0.000
Calditrichaeota	0.000	0.117	0.000	0.000	0.000
Acetothermia	0.000	0.078	0.000	0.000	0.000
Armatimonadetes	0.000	0.078	0.000	0.000	0.000
Asgardaeota	0.000	0.078	0.000	0.000	0.000
Atribacteria	0.039	0.000	0.000	0.000	0.000
Chrysiogenetes	0.000	0.000	0.000	0.000	0.038
Dependentiae	0.000	0.078	0.000	0.000	0.000
Diapherotrites	0.000	0.078	0.000	0.000	0.000
Elusimicrobia	0.000	0.000	0.000	0.000	0.038
Entotheonellaeota	0.000	0.000	0.049	0.000	0.000
Modulibacteria	0.000	0.078	0.000	0.000	0.000
WOR-1	0.000	0.078	0.000	0.000	0.000
Zixibacteria	0.000	0.078	0.000	0.000	0.000

Supplementary Table S4. Statistical values of the SH co-assembly, representing the five metagenomes.

**Contigs Stats**	SH Coassembly Contigs.db
Total Length	1,859,591,865
Num Contigs	994,545
Num Contigs > 2.5 kb	148,394
Num Contigs > 5 kb	31,221
Num Contigs > 10 kb	6,178
Num Contigs > 20 kb	870
Num Contigs > 50 kb	23
Num Contigs > 100 kb	1
Longest Contig	101,53
Shortest Contig	1
Num Genes (prodigal)	2,382,438
L50	281,061
L75	583,991
L90	817,245
N50	1,859
N75	1,305
N90	1,103
**Raw number of HMM Hits**	
Ribosomal_RNAs	227
Protista_83	2,396
Archaea_76	19,304
Bacteria_71	35,392
**Approx. number of genomes**	
eukarya (Protista_83)	3
archaea (Archaea_76)	9
bacteria (Bacteria_71)	356

Supplementary Table S6. Indexes of relative abundance and detection of each recovered MAG across the five SH metagenomes.

MAGs	Relative Abundance					Detection				
	H0	H1	H3	H4	H5	H0	H1	H3	H4	H5
SH-MAG111	0	0	0,72168791	0	0,27831209	0,00441	0,00554	0,99771	0,07316	0,95468
SH-MAG116	0	0	0,54889132	0,00013961	0,45096907	0,00295	0,00133	0,99243	0,12853	0,97785
SH-MAG12	0	0	0,54493692	0,11609335	0,33896972	0,0025	0,00193	0,99281	0,78714	0,9599
SH-MAG129	0	0	0,69848999	0,1220407	0,17946932	0,00313	0,02087	0,99531	0,74521	0,8047
SH-MAG130	0	0	0,38686291	0,14428399	0,4688531	0,00382	0,00389	0,97836	0,86299	0,98158
SH-MAG131	7,77E-06	0,99980086	0,0001669	0	2,45E-05	0,04034	0,99712	0,12015	0,02857	0,07146
SH-MAG143	0,90041192	8,62E-05	0,07427925	2,36E-05	0,02519905	0,99706	0,09119	0,89824	0,11717	0,62849
SH-MAG144	0	0	0,10728277	0,62320292	0,26951431	0,02051	0,01057	0,91627	0,99847	0,99011
SH-MAG148	0	0	0,00210879	0,72287575	0,27501545	0,00406	0,00258	0,23134	0,99751	0,97311
SH-MAG162	0	0	0,33797659	0,28950365	0,37251977	0,00712	0,0053	0,99119	0,99081	0,9919
SH-MAG169	0,53828526	0,38203929	0,04537899	4,69E-07	0,03429599	0,98488	0,84044	0,60269	0,03486	0,50812
SH-MAG192	0	0	0,3072886	0,22118822	0,47152318	0,00512	0,00153	0,98227	0,96535	0,99039
SH-MAG193	0	0	0,64640238	0	0,35359762	0,00147	0,00092	0,99165	0,05107	0,91743
SH-MAG29	0,99997034	0	5,39E-07	4,01E-06	2,51E-05	0,99993	0,00676	0,09412	0,03283	0,15348
SH-MAG3	0	0,00864616	0,78123545	0,00068058	0,20943781	0,0037	0,2329	0,99661	0,082	0,87538
SH-MAG78	0,00106885	0,0007131	0,73245797	0,00997231	0,25578776	0,07171	0,03031	0,9989	0,66337	0,99825
SH-MAG89	0	0	0,63255005	0,00085958	0,36659037	0,00305	0,00202	0,99812	0,22902	0,98909
SH-MAG93	0	0	0,48897198	0,15399497	0,35703306	0,00783	0,01217	0,98689	0,85548	0,96376
SH-MAG96	0	0	0,63205353	3,97E-06	0,36794251	0,00293	0,00202	0,99801	0,08462	0,98806

Supplementary Table S8. Presence and copy number of As resistance related genes in each analyzed MAG.

MAG ID	Gene presence and copy number						
	acr3	*arsA*	*arsC*	*arsH*	*arsJ*	*arsM*	*arsR*
**SH-MAG111**	1	1				1	2
**SH-MAG12**	1	1				1	2
**SH-MAG116**	2	1	1			1	2
**SH-MAG130**			1				2
**SH-MAG193**			1			1	2
**SH-MAG148**			2				3
**SH-MAG143**			1			1	3
**SH-MAG93**		1	1				2
**SH-MAG129**	1	1	3			1	3
**SH-MAG144**	1	1	1		1	1	1
**SH-MAG29**	1	3	1	1		2	3
**SH-MAG3**	1	3				1	3

Supplementary Table S3. Abundance and taxonomic classification of the 3801 ASVs detected in SH communities.

Supplementary Table S5. Statistical evaluation of enriched functional categories (SEED subsystem 1) for each of the five communities with respect to the other four (according to Welch's t-test).

Supplementary Table S7. Completion index of all detected KEGG modules in each analyzed MAG.
